# Technical Validation of a Multimodal Cognitive—Haptic Sudoku Platform Under Simulated Tremor Conditions

**DOI:** 10.3390/bioengineering12121340

**Published:** 2025-12-09

**Authors:** Calin Vaida, Oana Vanta, Gabriela Rus, Alexandru Pusca, Tiberiu Antal, Nicoleta Tohanean, Andrei Cailean, Daniela Jucan, Iosif Birlescu, Bogdan Gherman, Doina Pisla

**Affiliations:** 1 CESTER—Research Center for Industrial Robots Simulation and Testing, Technical University of Cluj-Napoca, 400641 Cluj-Napoca, Romania; calin.vaida@mep.utcluj.ro (C.V.); gabriela.rus@mep.utcluj.ro (G.R.); alexandru.pusca@mep.utcluj.ro (A.P.); tiberiu.alexandru.antal@mep.utcluj.ro (T.A.); bogdan.gherman@mep.utcluj.ro (B.G.); 2 European University of Technology, European Union; 3 Neurology I Department, Cluj-Napoca Emergency Clinical County Hospital, 400012 Cluj-Napoca, Romania; oana.vanta@elearn.umfcluj.ro (O.V.);; 4 Neurology Department, University of Medicine and Pharmacy “Iuliu Hatieganu”, 400012 Cluj-Napoca, Romania; 5 Technical Sciences Academy of Romania, B-dul Dacia, 26, 030167 Bucharest, Romania

**Keywords:** neurorehabilitation, Parkinson’s disease, Alzheimer’s disease, cognitive–motor therapy, haptic rehabilitation, dual-modal rehabilitation, sudoku-based cognitive training, gamified therapy

## Abstract

Neurological disorders such as Parkinson’s and Alzheimer’s diseases often involve overlapping motor and cognitive impairments that motivate integrated rehabilitation approaches. This study presents the technical validation of a dual-modality rehabilitation platform that combines haptic-based motor interaction with cognitive engagement through an adaptive Sudoku task in healthy adults under simulated tremor conditions. The system integrates a real-time tremor-filtering pipeline based on discrete wavelet denoising, Kalman smoothing, and wavelet packet decomposition, designed to attenuate high-frequency oscillations while preserving voluntary motion. The preclinical evaluation was carried out in two stages: (i) technical validation with healthy adults performing a standardized cognitive–haptic task under three conditions (no tremor, simulated tremor without filtering, simulated tremor with filtering) and (ii) extended usability testing with older participants without diagnosed neurological disorders. Quantitative evaluation focused on latency, performance degradation under simulated tremor, and partial restoration with filtering, while usability was assessed using the System Usability Scale (SUS). The platform achieved low end-to-end latency (41.4 ± 1.4 ms) and high usability (overall mean SUS = 81.4 ± 6.2), indicating stable performance and positive user feedback. Filtering significantly improved performance compared with unfiltered tremor but did not fully restore baseline performance, highlighting the current algorithm as a first-step compensation strategy rather than a complete solution. This work therefore demonstrates technical feasibility and interaction performance in healthy participants under simulated tremor; it does not assess clinical effectiveness and is intended to inform subsequent patient studies in populations with neurodegenerative diseases.

## 1. Introduction

The medical advancements of the last century have significantly increased life expectancy, but they have also led to a rise in age-related diseases [1,2]. Among these, neurodegenerative disorders like Alzheimer’s disease (AD) and Parkinson’s disease (PD) have become increasingly prevalent, posing significant burdens on healthcare systems and patients’ quality of life [3]. These conditions are characterized by the progressive deterioration of neurons, though they affect the brain in distinct ways and present different symptoms. 

In Alzheimer’s disease (AD), the accumulation of amyloid plaques and tau tangles leads to neuronal loss and brain atrophy, particularly in regions responsible for memory and cognition, causing progressive decline in memory, reasoning, and language [4], often accompanied by gradual motor impairment [5]. Motor symptoms in AD result from amyloid-β and tau pathology affecting extrapyramidal structures such as the basal ganglia, thalamus, and cerebellum, leading to tremor, gait instability, and parkinsonian features in advanced stages [6]. In Parkinson’s disease (PD), the degeneration of dopaminergic neurons in the substantia nigra disrupts motor control and produces tremor, rigidity, and bradykinesia, often followed by cognitive decline as the disease progresses [7]. Since both disorders share neurodegenerative mechanisms affecting motor and cognitive pathways, therapeutic strategies increasingly focus on combined interventions aimed at alleviating symptoms and slowing disease progression [8,9,10].

In recent years, technology-assisted rehabilitation has gained increasing attention as a complementary approach in neurodegenerative disease management. Cognitive rehabilitation, in particular, has been shown to help mitigate cognitive decline. Studies have demonstrated that engaging in cognitively stimulating leisure activities (CSLAs), such as Sudoku, can reduce the risk of Alzheimer’s disease and improve cognitive resilience. For instance, a study [11] found that individuals engaging in CSLAs exhibited slower cognitive decline, while another [12] demonstrated that Sudoku could serve as an effective tool for cognitive remediation, particularly in patients with prefrontal cortex dysfunction. Similarly, the authors of [13] reported that cognitive training programs in non-demented Parkinson’s patients led to moderate improvements in global cognitive status and working memory, with small but significant effects on verbal memory and executive function.

At the same time, neuromotor rehabilitation has evolved through the integration of robotic and haptic technologies, enhancing both motor and cognitive recovery [14].

Given the overlap between motor and cognitive deficits observed in neurological disorders such as Parkinson’s and Alzheimer’s diseases, integrated rehabilitation systems capable of addressing both domains simultaneously may offer more effective and comprehensive recovery strategies. Building upon these concepts, the present study introduces a dual-modal rehabilitation platform designed to combine motor and cognitive training within a single interactive framework. This study aims to address this gap by developing and testing a dual-modal rehabilitation system that combines.

• **Haptic-assisted motor training** using the Omega 7 device (Force Dimension, Nyon, Swirtzerland), equipped with a multi-stage tremor-filtering pipeline (wavelet denoising + Kalman smoothing) for real-time movement stabilization.
• **Cognitive stimulation** trough interactive Sudoku game with bilingual voice control (English/Romanian), adaptive difficulty, and performance logging for longitudinal tracking.
• **Personalized feedback**, including emotionally resonant auditory cues (e.g., familiar music/voice prompts) to enhance engagement in Alzheimer’s patients.

In this study, we focus strictly on preclinical feasibility in healthy adults using simulated tremor to examine interaction performance and system usability in a controlled setting. References to neurodegenerative conditions are provided as motivation for future clinical investigation rather than claims about tested efficacy.

Following the introduction, the paper is structured as follows: Section 2 describes the methodology, detailing the system architecture, haptic and voice-based interaction, cognitive components, and tremor simulation protocols. Section 3 presents the experimental results, including performance metrics under various conditions and the impact of tremor filtering. Section 4 offers a discussion on the system’s implications for integrated motor and cognitive rehabilitation. Finally, Section 5 concludes the paper and outlines directions for future clinical validation and system enhancements.

## 2. Related Work

Recent studies in neurorehabilitation have demonstrated that repetitive, feedback-guided robotic exercises significantly improve motor coordination and upper-limb function after stroke or neurological injury. These systems emphasize precision, repeatability, and real-time monitoring as key factors for measurable recovery outcomes, while haptic feedback enhances fine motor control and user engagement [15,16].

At the same time, hybrid or multimodal rehabilitation platforms combining robotic or haptic interfaces with cognitive components have begun to emerge, though their clinical translation remains limited. A recent systematic review on haptic technology in medicine highlighted that most applications remain in early experimental stages and rarely integrate simultaneous motor-cognitive tasks [17].

Tremor compensation has also been widely investigated in assistive and teleoperation systems. Classical approaches employ digital filtering, adaptive Kalman smoothing, and wavelet-based denoising to reduce involuntary oscillations without degrading voluntary motion [18,19]. More recent methods use machine-learning-based prediction to suppress physiological tremor while maintaining response speed in real-time control scenarios [20].

For the cognitive dimension, interactive puzzle or serious-game applications have been tested in older adults with mild cognitive impairment (MCI) or early Alzheimer’s disease, showing feasible improvements in attention, working memory, and task persistence [21]. Reviews of serious-game and virtual-reality-based interventions confirm their motivational value but also note that most systems lack physical interaction or haptic feedback, which limits their validity [22,23].

Overall, current literature supports the efficacy of robotic and computer-based interventions for either motor or cognitive rehabilitation, but integrated dual-domain systems remain underdeveloped. The present platform builds upon these findings by combining haptic interaction, adaptive tremor filtering, and cognitive task engagement into a unified, technically validated framework designed for Parkinson’s and Alzheimer’s disease rehabilitation.

## 3. Materials and Methods

The proposed system was designed as an enjoyable and complementary approach for individuals with combined motor and cognitive impairments, such as those encountered in neurodegenerative conditions. In this preclinical study, however, the platform is evaluated exclusively in healthy adults, with simulated tremor used to stress test interaction performance under controlled conditions. The long-term clinical target includes patients with Parkinson’s disease and Alzheimer’s disease, but no patients were enrolled in the present work.

To mitigate cognitive impairment—such as memory loss, confusion, and difficulty concentrating—the system integrates Sudoku, providing cognitive stimulation that enhances problem-solving skills and fosters independence, while reducing frustration through accessible and engaging gameplay.

At the same time, to support motor function and promote autonomy, the system incorporates the Haptic Omega 7 device as the primary controller. This allows users to navigate the Sudoku interface while performing hand exercises that improve fine motor skills and reinforce functional independence.

Additionally, the system features vocal command functionality for number input. This not only enhances the user experience by providing a hands-free alternative (reducing unnecessary strain from fine motor precision during this specific task) but also encourages verbal communication, improving social interaction and confidence, aspects often diminished in individuals with advanced neurodegenerative conditions such as Alzheimer’s disease or Parkinson’s disease [24].

**Figure 1:**
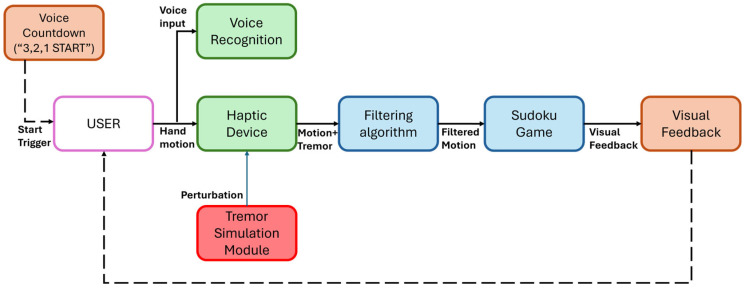
Overview of the experimental system showing the interaction between components. The haptic device captures hand motion, while the tremor simulation module adds perturbations. The filtering algorithm visually removes tremor before sending the signal to the Sudoku game, which integrates both haptic and voice inputs and provides visual feedback to the participant.

The overall system architecture, including hardware and software components, is illustrated in Figure 1.

The participant interacts with the system through two main input channels: hand motion captured by the haptic device and verbal commands processed by the voice recognition module. At the start of each trial, a dedicated voice countdown module delivers a brief auditory “3, 2, 1 START” signal to reduce freezing episodes often observed in Parkinsonian patients.

The haptic device transmits raw motion data containing both voluntary hand movement and artificial tremor introduced by the tremor simulation module. These combined signals are processed by the filtering algorithm, which visually removes tremor before sending the filtered motion to the Sudoku game environment. The Sudoku module integrates both filtered haptic input and recognized verbal commands, providing visual feedback to the participant in real time.

Importantly, the filtering is applied only at the visual level, meaning that while the participant still experiences the mechanically induced tremor in the hand and forearm, the cursor displayed on the screen moves smoothly. Although the system does not physically counteract the tremor, visually suppressing it prevents the patient from having to track a shaking cursor, reducing cognitive load and frustration. By allowing the patient to focus on intentional, controlled movements, the system facilitates more precise practice of motor tasks, similar to robotic rehabilitation approaches, thereby supporting neuroplasticity and contributing to long-term functional recovery.

### 3.1. Haptic Omega 7

The Omega 7, provided by Force Dimension [25] is a high-precision haptic device with 7 degrees of freedom (DOF)—6 DOF for motion, 1 DOF for active force feedback. It integrates high-resolution encoders and brushless DC motors, enabling precise force rendering up to 12 N with a positional resolution of <0.01 mm. The device provides 3 active translational DOF and 3 passive rotational DOF, allowing free wrist movement. Its grip-based interface supports controlled hand rehabilitation by applying adaptive force feedback.

Unlike conventional rehabilitation robots [26,27], which often focus on decoupled joint movements [28], the Omega 7 enables the movements presented in Table 1, as well as combinations of these movements. By integrating multiple degrees of freedom, the device allows patients to perform complex, multi-joint exercises that closely replicate real-life tasks [29,30]. This functional approach is particularly relevant for individuals with neurological motor impairments, as it directly addresses challenges such as rigidity, bradykinesia, and reduced coordination that can affect daily activities.

In this study, the Omega 7 was used exclusively as a 3D ergonomic input device, providing precise hand position tracking for cursor control. Although the device supports active haptic force feedback, no force or vibration output was delivered during the experimental sessions. This choice was intentional to focus on evaluating system usability and tremor filtering performance without introducing additional confounding feedback signals.

### 3.2. Sudoku

**Figure 2:**
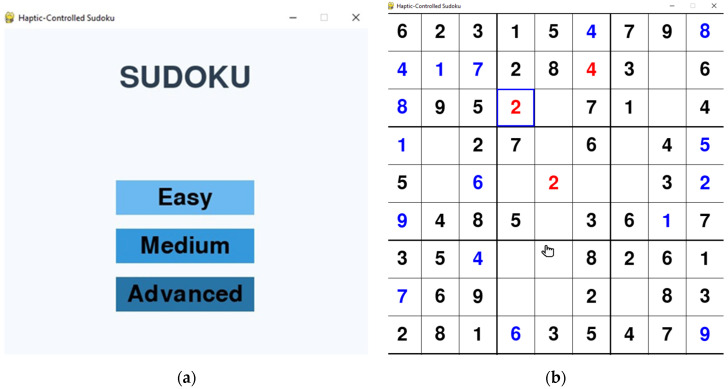
(**a**) Difficulty selection in the haptic Sudoku game. (**b**) Sudoku board with correct numbers in blue and incorrect ones in red.

The Sudoku game, developed in Python using Pygame (version 2.6.0), is designed as a motor-assisted cognitive training tool with adjustable difficulty levels (easy, medium, and hard-(Figure 2a), dynamically generating puzzles by removing 35, 45, or 55 numbers respectively from a valid 9 × 9 grid (Figure 2b)). Players interact with the game through the haptic device, which maps spatial coordinates to on-screen cursor movement, enabling grid navigation without requiring fine motor precision. The system implements a dual-language voice interface supporting both English (“one”, “two”) and Romanian (“unu”, “doi”) commands, specifically designed to assist older adult users who may lack English proficiency. Voice processing leverages Python’s speech_recognition library [31] with dedicated language configurations (en-US for English, ro-RO for Romanian), incorporating real-time validation against a precomputed solution matrix to ensure command accuracy. To minimize recognition errors, the system accepts only digit words from “one” to “nine” in English and “unu” to “nouă” in Romanian. Any other spoken input is automatically ignored, ensuring that only relevant commands are processed during gameplay.

Correct entries are retained while errors are flagged in red, providing immediate visual feedback. The game incorporates a “3, 2, 1, Start” countdown to mitigate freezing episodes—a Parkinson’s-specific feature that uses rhythmic auditory cues to initiate movement, a technique grounded in motor rehabilitation research [32]. Upon successful completion, the system triggers positive reinforcement messages and logs performance metrics (total completion time, Cell Completion Time (CCT), and number of input errors) to an Excel file for post-session analysis. Cell Completion Time (CCT) was defined as the interval from the first confirmed selection of the target empty cell to the first correct entry that finalizes (locks) that cell; if a different cell was selected and then abandoned, timing was bound to the final target cell only.

The system is explicitly designed for longitudinal assessment. All user interactions are automatically logged to a structured data file, capturing key performance metrics per session. These include, but are not limited to: total task duration, cell completion time, standard deviation of response times (as a measure of consistency), number of errors, and number of successfully completed cells. This granular data collection provides the foundation for tracking subtle changes in both motor performance and cognitive function over time.

### 3.3. Cognitive and Emotional Adaptation Features (For AD)

The system was designed to also support individuals with Alzheimer’s disease by incorporating emotionally meaningful stimuli. During each session, users can be exposed to familiar voice messages or preferred background music, enhancing emotional comfort and cognitive focus. These stimuli are user-specific and can be selected by caregivers or clinicians prior to therapy.

In the current implementation, familiar voice messages are recorded in advance by caregivers or clinicians using standard devices (e.g., smartphones or computers) and manually uploaded into a dedicated folder of the system. There is no fixed limit on the number of messages, but typically 5–10 short motivational or comforting phrases (e.g., greetings, encouragements, or reminders) are used, depending on the therapeutic plan. These recordings are played at predefined milestones, such as puzzle completion or successful error correction, to provide personalized and emotionally meaningful reinforcement. Similarly, preferred background music is handled through locally stored audio files (MP3 or WAV) selected by the caregiver. Playback is managed through the built-in Pygame audio module, which ensures offline operation without the need for external APIs or internet connectivity, thus maintaining data privacy and security. This customization aims to improve patient engagement and emotional comfort during cognitive exercises.

In addition, the system logs task performance data—such as error frequency, cell completion time, and interaction patterns—across sessions, with the goal of enabling longitudinal cognitive tracking. While a dynamic difficulty adjustment algorithm is currently in development, the current design supports manual selection of puzzle difficulty to match the user’s cognitive capabilities.

### 3.4. Methodology for Preclinical Testing

The preclinical testing phase was conducted in two consecutive stages, involving two participant groups:

(1) A technical validation group, initially composed of healthy adults;
(2) An extended group, including additional participants without diagnosed neurological disorders, representing the broader usability evaluation phase.

For clarity, these groups will hereafter be referred to as the technical validation group and the extended group throughout the manuscript.

Testing was first carried out on the technical validation group to verify the technical performance, stability, and safety of the system under controlled laboratory conditions. Based on these results, the protocol was subsequently extended to older adults, to assess usability, tolerability, and interaction comfort in realistic conditions reflecting age-related motor and cognitive characteristics.

Although elderly individuals were included, the study does not constitute a clinical trial, as it did not involve therapeutic intervention or the assessment of clinical outcomes. Instead, it represents a technical and functional validation stage, intended to confirm that the proposed system operates safely and effectively in its target user population and can serve as a foundation for future controlled clinical studies.

All procedures were approved by the Ethics Committee of the Technical University of Cluj-Napoca (Approval No. UTCN-S01-02.06.2025 and UTCN-S02-06.10.2025) and conducted according to institutional research standards and the 1964 Declaration of Helsinki. All participants provided written informed consent and were informed of their right to withdraw at any time. As the study involved minimal risk, no financial compensation or additional provisions were necessary.

#### 3.4.1. Participants and Study Design

- **Technical validation group**

A total of 15 healthy adults (age range: 25–45 years) were enrolled in this preclinical feasibility study. Participants were directly invited from the university community, including students, faculty members, researchers, and collaborating clinicians. This targeted approach ensured adherence to the study protocol in a controlled environment and minimized variability due to external clinical factors.

All interested individuals received detailed information about the study’s objectives and procedures and were screened using predefined eligibility criteria. Inclusion required prior experience with Sudoku and a basic understanding of its rules, while exclusion criteria included neurological disorders, significant visual impairments, or musculoskeletal conditions affecting hand function. The first 15 eligible individuals who provided written informed consent were selected.

Healthy participants were deliberately chosen to allow a controlled evaluation of the system’s technical performance and usability prior to extending the platform to clinical populations with neurological disorders. For consistency in cognitive task performance and to minimize learning bias, only the “easy” Sudoku level (removal of 35 digits) was used in all tests to ensure fast task execution during motor interaction assessment.

Given the exploratory preclinical nature of this work, no formal a priori power calculation was performed. Instead, the sample size for the technical validation cohort was determined pragmatically. We targeted N = 15 healthy participants, in line with previous preclinical haptic and neurorehabilitation studies using within-subject designs to detect large effects with acceptable power. In repeated-measures ANOVA, the required sample size is typically smaller than in an equivalent between-subject design when effects are large and within-subject variability is low. Accordingly, the present analyses should be interpreted primarily as feasibility results, with emphasis on effect sizes (η^2^, Cohen’s d) and consistent patterns across outcomes rather than on marginal *p*-values.

- **Extended group**

To further evaluate the usability and comfort of interaction with the proposed system, an additional testing phase was conducted with elderly participants, representing the target demographic for future deployment of the cognitive–haptic platform. A total of 12 older adults (age range: 60–80 years) were included in this phase.

Participants were community-dwelling individuals without diagnosed neurological, visual, or musculoskeletal disorders that could affect task performance. The selection of participants was motivated by the system’s intended use in rehabilitation and cognitive training programs designed primarily for older adults. Their inclusion allowed the research team to identify potential ergonomic, perceptual, or cognitive barriers that may not be observable in younger healthy users.

All participants performed the same Sudoku-based task as in the previous phase, combining haptic and voice interaction. The test sessions focused exclusively on usability, ease of interaction, and user comfort. No quantitative experimental comparison or inferential statistical analysis (e.g., ANOVA) was applied, as the purpose of this phase was descriptive and exploratory.

Each participant completed a standardized System Usability Scale (SUS) questionnaire and a short, structured interview at the end of the session. Feedback was analyzed qualitatively to identify common patterns related to task complexity, fatigue, visual clarity, and intuitiveness of control.

Testing sessions were conducted under the same conditions as in the technical validation phase, with each session lasting approximately 30–45 min. Rest breaks were provided as needed, and all participants gave written informed consent prior to participation.

The aim of this extended phase was to assess whether older adults, whose age range is closer to the intended clinical population, would experience any major difficulties when interacting with the platform. In this context, the absence of substantial usability problems or task failures was interpreted as an encouraging indication that the system can be used safely and comfortably by older adults, rather than as a dataset intended for detailed modeling of age-related performance differences.

#### 3.4.2. Experimental Procedure and Data Collection

**Figure 3:**
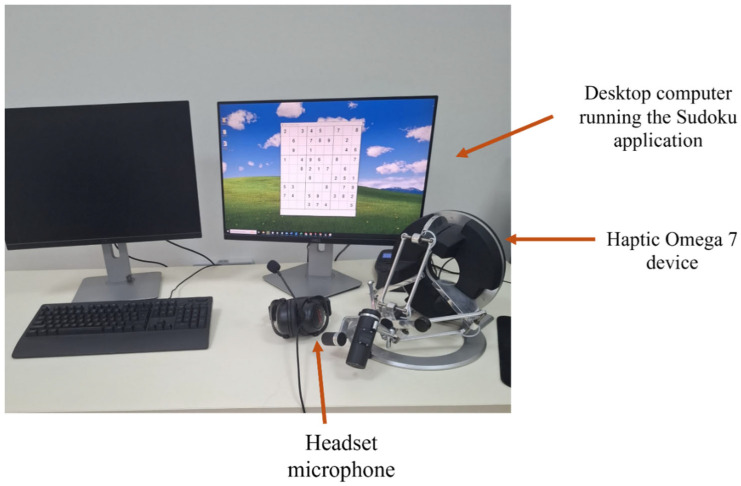
Experimental setup with haptic control for Sudoku.

The experimental setup, presented in Figure 3, consisted of

• The Haptic Omega 7 device—mounted on a stable desk, allowing for free and comfortable upper limb movements.
• A desktop computer running the custom-developed Sudoku application.
• A headset microphone, positioned close to the user’s mouth to ensure clear audio capture.

The experiments were conducted in a typical laboratory environment with background conversations and ambient noise present, reflecting realistic conditions rather than a controlled sound-isolated setup. Before starting each testing session, participants received a standardized overview of the system and a short hands-on familiarization period with the interface and controls. Testing sessions lasted approximately 30–45 min, covering both the system interaction phase and post-task feedback collection.

The testing phase aimed to evaluate the system’s performance and usability in the extended group, focusing on ease of interaction, response consistency, and perceived comfort during haptic and voice control.

For every session, the system automatically logged detailed performance data—including total completion time, Cell Completion Time (CCT; see Methods for definition), error counts, and response-time variability—enabling quantitative tracking of user performance. Particular attention was given to the system’s responsiveness during motor task execution, the accuracy of voice commands for number input, and the subjective user experience during prolonged use.

Usability was evaluated using the System Usability Scale (SUS), a standardized and validated 10-item questionnaire widely adopted in human–computer and human–robot interaction studies. The goal was to identify potential areas for improvement in interface design, task flow, and interaction efficiency, based on both objective performance data and standardized subjective feedback.

### 3.5. Data Analysis

The statistical analysis reflected the sequential validation process of the study, addressing both the technical validation and extended usability phases. For the cohort, a within-subject design was employed, in which each participant performed the cognitive-haptic task under three controlled experimental conditions: (1) no tremor, (2) simulated tremor without filtering, and (3) simulated tremor with filtering. This setup enabled the controlled evaluation of the system’s response to tremor perturbations and the quantitative validation of the visual filtering algorithm.

Descriptive statistics (mean, standard deviation, and 95% confidence intervals) were computed for each condition and outcome variable:

- Cell Completion Time;
- Total puzzle completion time;
- Number of input errors.

A one-factor repeated-measures analysis of variance (ANOVA) [33] was performed to test for differences among the three experimental conditions. When the overall F-test was significant, Bonferroni-adjusted pairwise comparisons were applied to determine specific condition differences while controlling for family-wise error.

Assumption checks were performed to validate statistical results. Normality of residuals was assessed visually using Q-Q plots. Homogeneity of variance and sphericity were evaluated using residuals-vs-fitted plots and Mauchly’s test, respectively. When sphericity assumptions were violated, the Greenhouse–Geisser correction was applied to adjust degrees of freedom and ensure robust *p*-values [34]. A significance threshold of α = 0.05 was used for all tests, and effect sizes were reported using partial eta squared (η^2^) or Cohen’s d, providing standardized measures of effect magnitude.

The three outcome measures (Cell Completion Time, total puzzle completion time, and number of input errors) were pre-specified as primary endpoints for this preclinical study. Given this and the exploratory nature of the work, no additional correction was applied across outcome domains; instead, we focus on the convergence of results and report effect sizes for all comparisons.

Mauchly’s test did not indicate significant violations of sphericity for any of the repeated-measures ANOVAs. For completeness, analyses were repeated with Greenhouse–Geisser corrections; the pattern of significant and non-significant contrasts remained unchanged, and therefore unadjusted degrees of freedom are reported.

All statistical analyses were performed using Python (version 3.11.9) with the statsmodels package (version 0.14.2) for ANOVA and post hoc computations. Diagnostic visualizations (Q–Q plots and residuals-vs-fitted plots) were generated using Matplotlib (version 3.9.2) and Seaborn (version 0.13.2). Results were cross-validated using R (version 4.3.3) with the afex and emmeans packages to ensure reproducibility and transparency.

### 3.6. Tremor Simulation for Preclinical Technical Assessment

To ensure system readiness prior to usability testing with the extended group, a preclinical procedure was implemented to simulate hand tremor in healthy volunteers. This stage enabled the calibration and validation of the system’s capability to detect, process, and filter tremor-induced perturbations under reproducible laboratory conditions.

#### 3.6.1. Equipment and Experimental Setup

**Figure 4:**
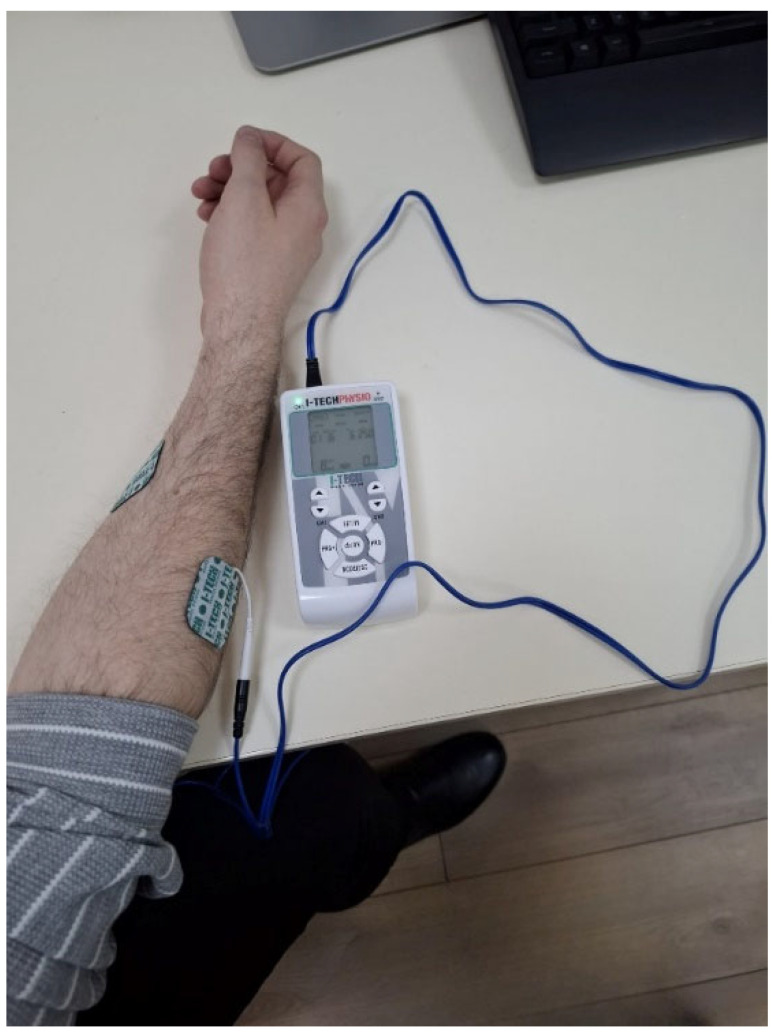
Electrode placement on the forearm.

The simulation of tremor was conducted using the I-Tech Physio, a neuromuscular stimulation device manufactured by I-TECH Medical Division [35]. Surface electrodes were positioned on the flexor carpi radialis and extensor carpi radialis longus [36] of the participant’s forearm Figure 4. The selection of these muscle groups was made to induce involuntary oscillatory contractions in the frequency range typically reported for resting tremor in Parkinson’s disease [37], while acknowledging that peripheral electrical stimulation does not reproduce the central pathophysiological mechanisms of Parkinsonian tremor.

The participants from the technical validation group (aged 25–45 years) were included in this experimental phase. The tremor was induced under controlled stimulation protocols designed to replicate different tremor profiles.

#### 3.6.2. Stimulation Protocol

The simulation parameters were selected to replicate key characteristics of Parkinsonian tremor, typically reported between 6 and 8 Hz [38]. Two representative frequencies were used: 6 Hz, corresponding to the most frequent resting tremor, and 8 Hz, representing the upper bound observed in severe cases.

Transcutaneous electrical stimulation was applied to the participant’s forearm using the following parameters:

Frequency: 6 Hz or 8 Hz (depending on the protocol).

Pulse width: 200 μs.

Current intensity: 10 mA.

Session duration: 4 min.

The induced oscillatory muscle activity generated controlled perturbations in the user’s motor input, reducing cursor stability without causing discomfort. This configuration allowed evaluation of the system’s tremor-detection and real-time filtering performance under repeatable preclinical conditions.

It is important to emphasize that this simulated tremor model does not reproduce other cardinal motor symptoms of Parkinson’s disease, such as bradykinesia, rigidity, or freezing of gait and upper-limb movement. The induced oscillations reflect only a peripheral approximation of tremor-like activity, and therefore the present results should not be interpreted as algorithm performance in actual pathological tremor.

The preclinical stimulation setup was reviewed and validated by clinical neurologists to ensure that the selected frequencies and performance metrics accurately reflected relevant pathological tremor characteristics.

#### 3.6.3. Tremor Signal Acquisition

The tremor induced by neuromuscular stimulation was measured using the force sensors integrated into the Haptic Omega 7 device. The sensors recorded real-time force data along the X and Y axes, allowing for the analysis of tremor amplitude and frequency.

The raw force data were collected at a sampling rate synchronized with the haptic system control loop, ensuring accurate detection of oscillatory movement components. The dominant tremor frequency was confirmed to be consistent with the stimulation protocol, particularly at 6 Hz during the primary stimulation phase.

#### 3.6.4. Filtering

**Figure 5:**
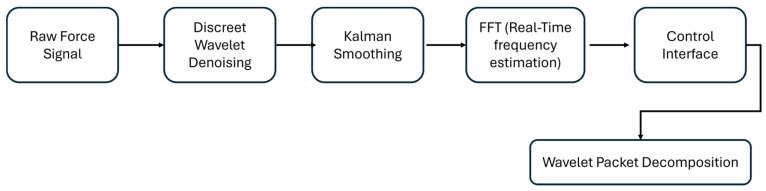
Real-time tremor filtering pipeline.

The real-time filtering algorithm was developed to suppress tremor-related oscillations while preserving voluntary motion during haptic interaction. As illustrated in Figure 5, the multi-stage signal-processing pipeline operates on the force data acquired from the Haptic Omega 7 sensors and consists of six main stages.

- **Step 1—Raw Force Signal Acquisition**

Continuous force data are collected along the *X* and *Y* axes, containing both voluntary movement components and high-frequency perturbations. These raw signals serve as the input for the filtering pipeline.

- **Step 2—Discrete Wavelet Denoising**

A Discrete Wavelet Transform (DWT) is applied to remove high-frequency sensor noise while preserving meaningful motion components [39]. The Daubechies 4 (db4) wavelet was selected due to its balance between smoothness and localization properties. Noise reduction is achieved through soft-thresholding of the detail coefficients using the universal threshold *λ* computed as

(1) $$\lambda =\sigma \sqrt{2\cdot \ln\left(N\right)}$$

where *σ* represents the estimated noise standard deviation, calculated as

(2) $$\sigma =\frac{median\left(\left|D_{j}\right|\right)}{0.6745}$$

In Equation (1), *N* denotes the signal length, and *D_j_* represents the set of first-level detail coefficients. The constant 0.6745 normalizes the median absolute deviation assuming Gaussian noise [40].

- **Step 3—Kalman Smoothing**

The denoised signal is further processed using a Kalman filter to smooth temporal variations and suppress residual measurement noise. The filter follows the standard linear state-space formulation:

(3) $${\hat{x}}_{k\left|k\right.}=A\cdot {\hat{x}}_{k-1\left|k-1\right.}$$

(4) $$K_{k}=\frac{P_{k\left|k-1\right.}\cdot H^{T}}{H\cdot P_{k-1\left|k-1\right.}\cdot H^{T}+R}$$

(5) $${\hat{x}}_{k\left|k\right.}={\hat{x}}_{k\left|k-1\right.}+K_{k}\left(z\cdot k-H\cdot {\hat{x}}_{k\left|k-1\right.}\right)$$

where *A* = 1 is the state transition matrix describing the evolution between time steps, *K_k_* is the Kalman gain, *P* the predicted error covariance, *R* the measurement noise covariance (set to 0.1), and *Q* the process noise covariance (set to 0.01). This stage ensures robust low-latency estimation of the voluntary trajectory.

- **Step 4—Frequency Analysis**

A Fast Fourier Transform (FFT) is applied to short, overlapping time windows (2 s, 50% overlap) of the processed signal. The frequency bin with the highest amplitude identifies the dominant oscillation frequency (*f_dom_*), allowing dynamic adaptation of filtering parameters in real time. The FFT is also applied offline to recorded data for validation and quantification of tremor attenuation.

The FFT is defined as

(6) $$X\left(k\right)=\sum_{n=0}^{N-1}x\left(n\right)\cdot e^{\frac{-j\cdot 2\pi \cdot k\cdot n}{N}}$$

The dominant frequency is computed as

(7) $$f_{dom}=\frac{k_{peak}\cdot f_{S}}{N}$$

where *F_s_* = 50 Hz is the sampling frequency and *k_max_* the index of the maximum amplitude in the magnitude spectrum.

- **Step 5—Wavelet Packet Decomposition (WPD)**

Wavelet Packet Decomposition is employed to separate voluntary and involuntary motion components based on their frequency content. Unlike the fixed 6 Hz cutoff, the algorithm adaptively adjusts the tremor attenuation threshold according to the dominant tremor frequency $f_{d}$ identified in Step 4.

Specifically, WPD nodes corresponding to frequency bands above $f_{d}-1\;\mathrm{Hz}$ are classified as tremor-dominant and attenuated, while lower-frequency nodes are reconstructed to preserve voluntary motion. This adaptive boundary ensures robustness across users and sessions, accounting for variations in tremor frequency over time.

The reconstructed signals follow the general form:

(8) $$x\left(t\right)=\sum_{i=1}^{N}c_{i}\cdot \phi_{i}\left(t\right)$$

where $c_{i}$ are the wavelet packet coefficients and $\phi_{i}(t)$ the corresponding basis functions. By coupling WPD with real-time FFT analysis, the filter dynamically adapts to changes in tremor characteristics, providing fine-grained separation between voluntary and involuntary motion while maintaining real-time performance.

- **Step 6—Control Interface Integration**

The filtered control signal is forwarded to the main system interface, where it drives real-time interaction and visual feedback synchronization. The control loop ensures stable, low-latency operation of the haptic interface, maintaining the natural responsiveness of the task.

Each preprocessing stage in the pipeline addresses a specific challenge:

- Wavelet denoising eliminates high-frequency noise while preserving motion dynamics.
- Kalman smoothing provides optimal trajectory estimation under jitter and latency.
- WPD isolates tremor components adaptively in the frequency domain.

The algorithm was implemented in Python and integrated into the system’s control architecture, operating synchronously with the data acquisition module at a 50 Hz sampling rate. This configuration allows the processing of raw force data, application of the full filtering pipeline, and continuous update of the control interface without perceptible delay.

**Figure 6:**
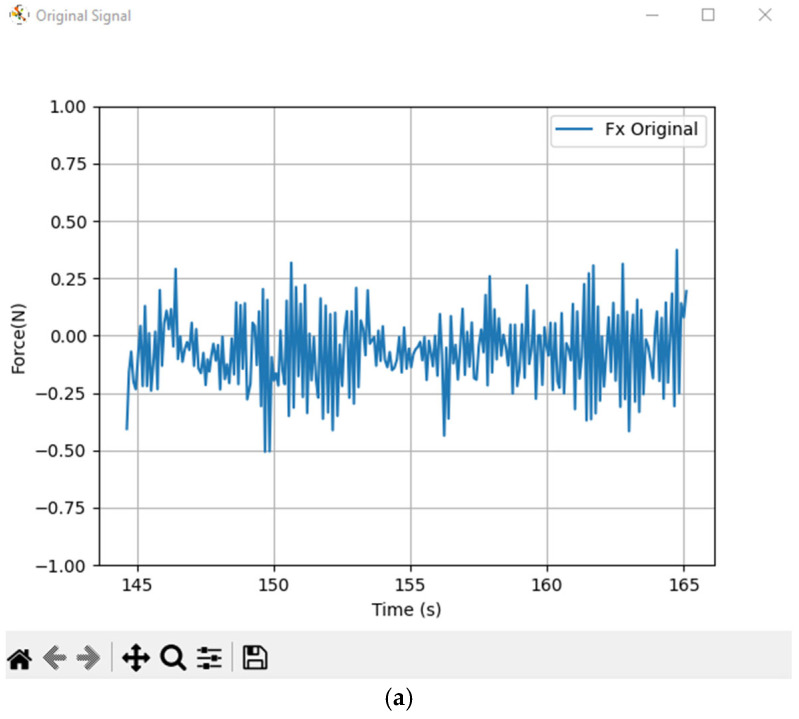

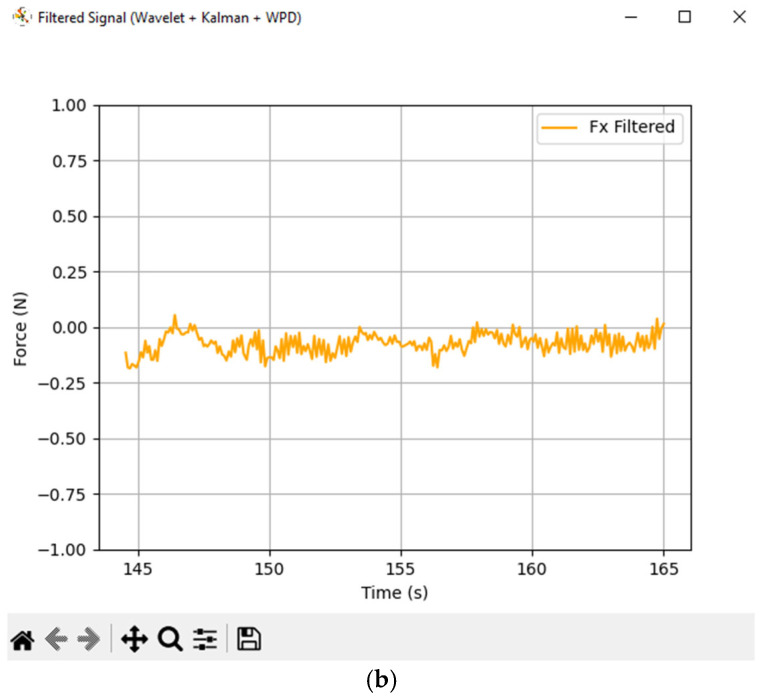
(**a**) Raw force signal along the *X*-axis. (**b**) Filtered signal using Wavelet, Kalman, and WPD methods.

Figure 6a,b illustrate the force signal along the X axis of the haptic device, before and after filtering, selected for clarity as representative of tremor-induced oscillations. The horizontal axis shows time in seconds, while the vertical axis represents force in Newtons (N), measured by the device’s built-in sensors and used to monitor tremor frequency through spectral analysis.

## 4. Results

The results are presented for the preclinical validation stages. Analyses first address the healthy cohort, used for technical validation of the tremor-filtering algorithm under controlled conditions. The extended (older adult) group is then summarized for usability only.

### 4.1. Technical Validation

**Figure 7:**
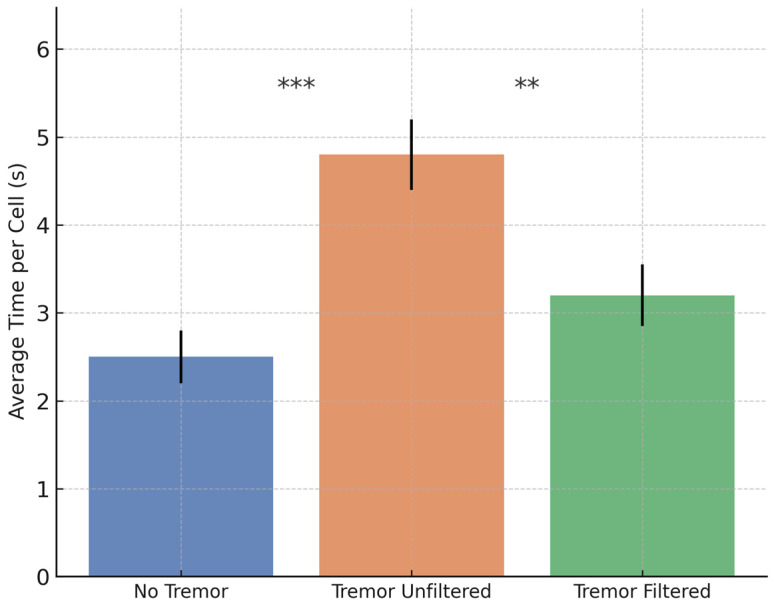
Mean ± 95% CI for cell completion time across the three experimental conditions: no tremor, tremor unfiltered, and tremor filtered. Asterisks indicate Bonferroni-adjusted significant pairwise differences.

**Figure 8:**
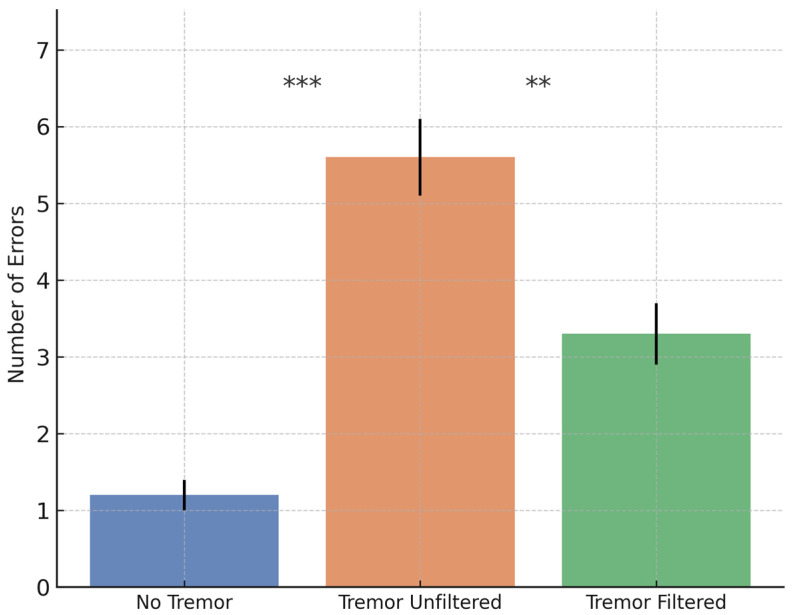
Mean ± 95% CI for the number of input errors across conditions. Tremor significantly increased errors, while filtering reduced them, though not to baseline levels. Asterisks indicate Bonferroni-adjusted significant pairwise differences.

**Figure 9:**
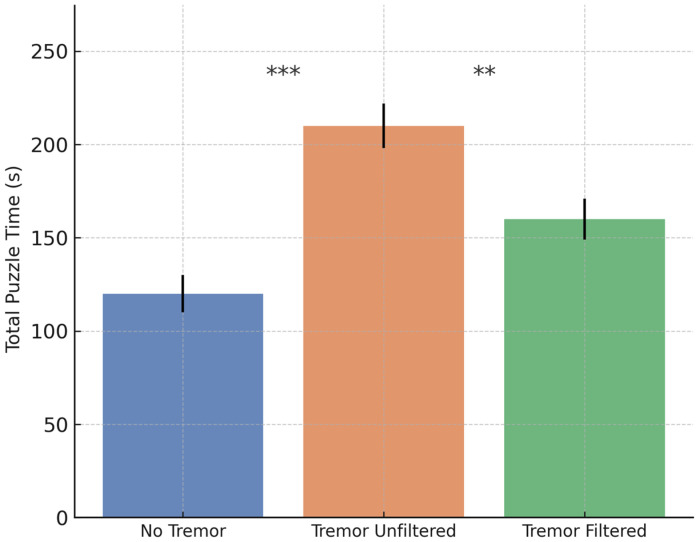
Mean ± 95% CI for total puzzle completion time across conditions, mirroring the pattern observed for cell completion time. Bonferroni-adjusted significance is indicated by asterisks.

To evaluate the system’s performance and filtering efficiency under controlled conditions, a comparative analysis was conducted on the Healthy cohort. Each of the 15 participants completed the cognitive–motor task under three predefined conditions: (1) baseline without tremor, (2) simulated tremor without filtering, and (3) simulated tremor with active filtering. This design enabled a systematic assessment of both the impact of tremor on task performance and the effectiveness of the filtering algorithm in restoring stability and accuracy. Group means with 95% confidence intervals are presented in Figure 7, Figure 8 and Figure 9. Statistical comparisons were based on a repeated-measures ANOVA with Bonferroni-adjusted post hoc tests, as detailed in Section 3.5.

For Cell Completion Time, a significant main effect of Condition was found, F(2, 28) = 32.45, *p* < 0.001, η^2^ = 0.70. Pairwise comparisons showed that unfiltered tremor significantly increased task time relative to baseline (*p* < 0.001), while filtering reduced it compared to the unfiltered condition (*p* = 0.002). Performance in the filtered condition remained slower than baseline (*p* = 0.021).

A similar trend was observed for the number of input errors, where the ANOVA showed a significant effect of Condition, F(2, 28) = 45.87, *p* < 0.001, η^2^ = 0.77. The unfiltered tremor condition produced significantly more errors than baseline (*p* < 0.001). Filtering reduced the error rate compared to the unfiltered condition (*p* < 0.001), though errors remained slightly higher than baseline (*p* = 0.048).

For total puzzle completion time, the ANOVA indicated a main effect of Condition, F(2, 28) = 28.62, *p* < 0.001, η^2^ = 0.67. Completion time increased significantly under unfiltered tremor (*p* < 0.001) and improved with filtering (*p* = 0.003), although it remained higher than baseline (*p* = 0.035).

Taken together, these results indicate that the filtering pipeline partially, but not completely, restores baseline performance. All three primary outcomes remained significantly worse than in the no-tremor condition, quantifying the residual impact of simulated tremor on task execution even after compensation. The current implementation should therefore be interpreted as a first-step tremor attenuation strategy rather than as a full restoration of voluntary motion.

Model assumptions for the ANOVA were verified through residuals-vs-fitted and Q–Q plots. These visual checks confirmed approximate normality and identified only mild heteroscedasticity, which is common in small-N preclinical studies and did not alter the pattern of significant pairwise results.

### 4.2. System Latency Analysis

To confirm the suitability of the rehabilitation platform for real-time cognitive–motor interaction, a latency analysis was conducted following the integration of the tremor-filtering pipeline into the control architecture. The system operated at a fixed sampling rate of 50 Hz, with all filtering operations—Discrete Wavelet Transform, Kalman smoothing, and Wavelet Packet Decomposition—executed in real time within the main control loop.

Latency was defined as the elapsed time between the acquisition of force data from the haptic device and the corresponding visual update on the user interface, encompassing signal processing, decision logic, and rendering. Measurements were recorded continuously using time stamps synchronized with the control loop during active tremor conditions.

**Figure 10:**
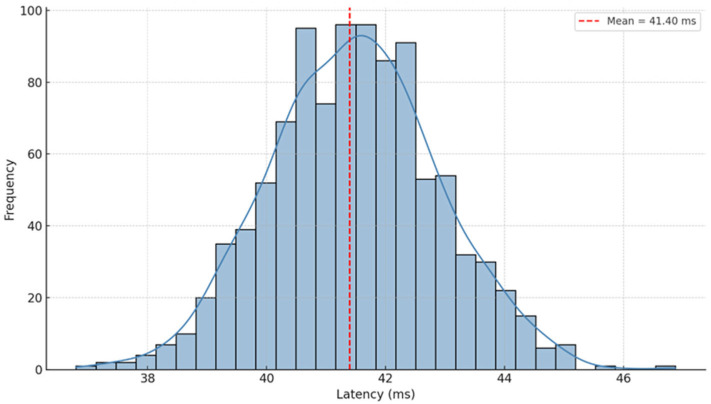
Latency distribution of the real-time control system with a mean of 41.40 ms.

Across all test sessions, the mean system latency was 41.40 ± 1.42 ms, with a minimum of 39.86 ms and a maximum of 44.86 ms (Figure 10). These values demonstrate that the multi-stage filtering pipeline maintains low and consistent latency despite its computational complexity.

Latency remained well below perceptual thresholds typically associated with interaction lag (≈100 ms), ensuring that user actions and visual feedback were perceived as continuous. The low variability further confirms the stability of the processing pipeline, supporting smooth, uninterrupted task execution during cognitive–motor rehabilitation activities.

### 4.3. System Usability and Subjective Feedback

**Figure 11:**
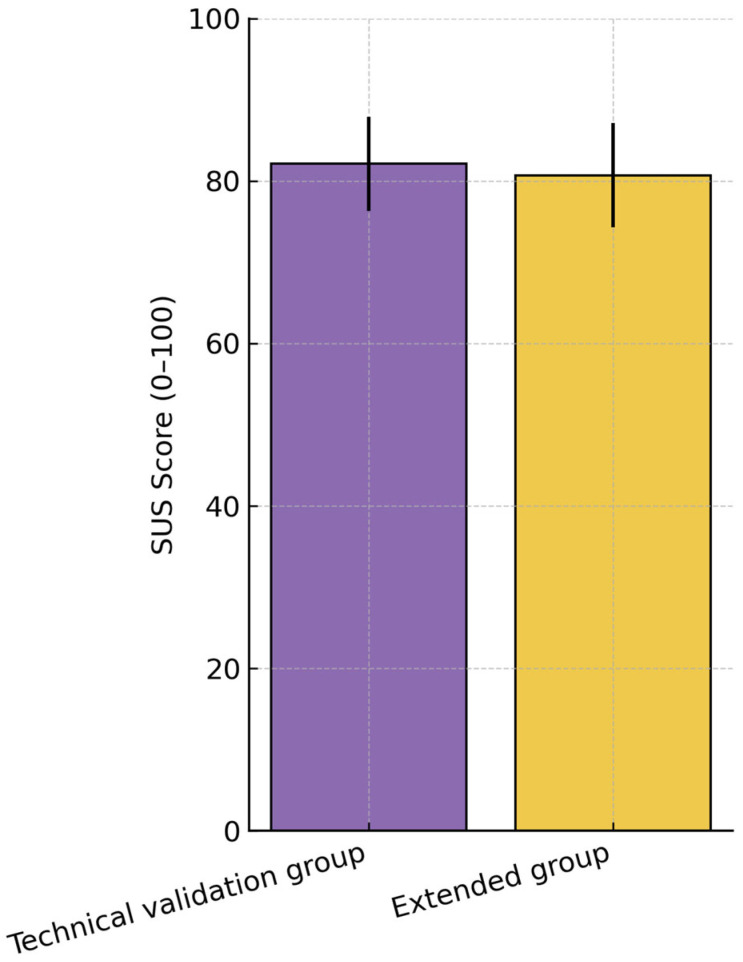
Mean ± SD System Usability Scale (SUS) scores for the technical validation group and the extended group.

User experience was evaluated across all participant groups, focusing on perceived comfort, system responsiveness, ease of use, and overall usability. The System Usability Scale (SUS) was completed at the end of the testing procedure, with scores ranging from 0 (poor usability) to 100 (excellent usability). SUS scores in the extended group of older adults were comparable to those observed in the technical validation group (Figure 11), with both cohorts scoring well above the conventional 70-point threshold for “good” usability. All older participants were able to complete the full interaction protocol without dropouts or task breakdowns. Qualitative feedback indicated that, after a short familiarization period, older adults felt comfortable with the haptic device and the Sudoku interface and did not report major usability issues, pain, or excessive fatigue. Several participants spontaneously remarked that the task was “engaging” and “pleasantly challenging”, suggesting that the platform is acceptable and motivating also in an age range closer to the intended clinical population. The overall mean SUS score across participants was 81.4 ± 6.2, which corresponds to a high usability level according to standardized SUS interpretation guidelines.

Participants described the system as intuitive, responsive, and easy to learn after a short familiarization period. Minor coordination difficulties between haptic input and on-screen feedback were quickly overcome, and no discomfort, excessive cognitive effort, or fatigue was reported during use.

As illustrated in Figure 11, both the technical validation and extended groups reported consistently high usability scores, confirming that the system maintained accessibility and ease of interaction throughout all testing stages.

The overall SUS scores (Figure 11) are expressed on the standard 0–100 scale, which results from converting the raw item responses into a composite usability index. This standardized scale allows for direct comparison between studies and across testing stages.

**Figure 12:**
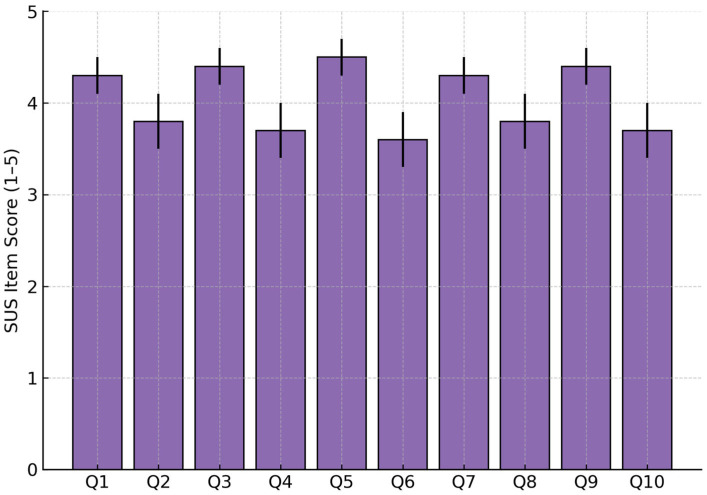
Mean ± SD per SUS item (Q1–Q10).

Figure 12 presents the average ratings for each SUS item (Q1–Q10). Questions related to ease of use, task integration, and user confidence received the highest scores, while those addressing system complexity and consistency scored slightly lower, indicating potential directions for future ergonomic optimization.

## 5. Discussion

In this study, a cognitive–haptic platform was developed and tested under preclinical conditions to assess system performance, stability, and user interaction. The validation process was organized in two consecutive stages—technical validation and extended usability testing—focused on evaluating latency, force response, and user adaptability during a standardized cognitive–motor task. The experimental setup allowed continuous monitoring of interaction dynamics, confirming that the haptic and vocal subsystems operated synchronously and without perceptible interference.

This study was conducted in healthy participants under simulated tremor and therefore does not establish clinical effectiveness for patients with neurodegenerative diseases. The observed effects are limited to task-level interaction metrics in a controlled environment. Generalization to clinical populations requires dedicated trials in representative cohorts and settings. Accordingly, any references to Parkinson’s and Alzheimer’s diseases throughout the manuscript should be interpreted strictly as clinical motivation and design context, not as claims about tested efficacy in these populations.

The recorded data indicated that the system maintained consistent latency and force stability across all test sessions. The integration of haptic control with a cognitive task enabled an effective assessment of user adaptation to multimodal feedback, revealing low variability in motor precision and task completion time. Usability analysis through the System Usability Scale (SUS) demonstrated that participants rapidly became familiar with the interface and maintained stable performance. Minor differences between technical and extended testing phases reflected natural variation rather than design limitations.

In addition, the tremor perturbations used in this study were generated by peripheral neuromuscular stimulation in healthy volunteers. This approach reproduces oscillatory components in the 6–8 Hz range but not the central neurophysiological mechanisms of Parkinsonian tremor. Core clinical features such as bradykinesia, rigidity, and freezing were absent, and the filtering algorithm has not yet been tested against genuine pathological tremor. Future work will therefore need to evaluate the pipeline directly in patients with neurodegenerative diseases before any claims about clinical tremor compensation can be supported.

Another limitation concerns the evaluation of the filtering pipeline. In this preclinical study, we restricted the analysis to task-level behavioural measures and time-domain inspection of force and position traces, as our primary focus was on interaction feasibility and usability rather than on an exhaustive characterization of the signal-processing algorithm. Consequently, we did not perform a detailed frequency-domain analysis of the attenuation profile of the 6–8 Hz components.

Although the current validation was limited to non-clinical participants and a controlled environment, the findings demonstrate that the platform reached a sufficient maturity level for safe extension toward clinical testing. Further validation is planned to explore performance in subjects with neurological impairments, focusing on usability, tremor filtering efficiency, and task reliability under medical supervision. To support this transition, a preliminary clinical protocol has been defined to establish the framework for upcoming validation under clinical supervision.

**Planned Clinical Validation Protocol**

**Objective**. To evaluate the clinical usability, safety, and interaction performance of the cognitive–haptic platform in patients diagnosed with Parkinson’s disease and Alzheimer’s disease.

**Study design**. Prospective, within-subject, single-session pilot study conducted jointly with neurology and rehabilitation specialists. The testing procedure will replicate the Sudoku-based cognitive–motor task from the preclinical phase, integrating haptic and vocal interaction to assess coordination and response consistency.

**Participants**. Adult volunteers with confirmed PD or AD diagnosis, able to perform controlled upper-limb movements and follow verbal instructions. Inclusion and exclusion criteria will comply with clinical safety standards and institutional ethics regulations.

**Procedure**. Each participant will complete a familiarization step followed by a 30–40 min standardized task involving calibration, interactive task execution, and post-session evaluation through the System Usability Scale (SUS) and clinician interview.

**Data collection**. Force and position data will be logged at 1 kHz and 200 Hz, respectively, synchronized with timestamps for voice inputs and task events. Quantitative indicators such as completion time, latency, and accuracy will be analyzed together with usability feedback.

**Ethics**. The pilot will be conducted under institutional ethical approval, following the Declaration of Helsinki, with informed consent obtained prior to participation.

One relevant aspect of the system is its dual functionality, combining motor interaction through the Omega 7 haptic interface with a structured cognitive task based on an adaptive Sudoku module. While most rehabilitation systems are designed to address either motor [9,10,11] or cognitive training separately [13,14,15,16,17], this configuration allows both domains to be activated in parallel. The simultaneous engagement of motor control and problem-solving tasks promotes more complex user interaction and provides a richer dataset for evaluating coordination, attention, and error dynamics during task execution.

## 6. Conclusions

The study presented a cognitive–haptic platform designed for combined motor and cognitive interaction, integrating a haptic interface with a structured Sudoku-based task. The preclinical evaluation provided consistent results in terms of latency, stability, and usability, indicating that the system architecture performs reliably under controlled conditions. Both the technical validation and extended usability phases highlighted stable response behavior and high user acceptance, confirming that the platform can support fine-motor interaction tasks with minimal fatigue.

The results suggest that the system has reached a sufficient level of technical maturity to progress toward clinical validation. A preliminary protocol has been defined for testing the platform with participants diagnosed with Parkinson’s disease and Alzheimer’s disease, focusing on usability, safety, and interaction reliability under medical supervision.

One relevant aspect of the system is its dual functionality, which combines motor interaction through the Omega 7 haptic interface with cognitive problem-solving through the Sudoku module. This integration enables simultaneous activation of motor and cognitive domains, promoting complex user engagement and generating comprehensive datasets for analyzing coordination, attention, and task-related errors.

Findings should be interpreted as evidence of technical feasibility and usability under simulated tremor in a preclinical context, not as proof of clinical benefit.

Future developments will address adaptive task difficulty, longer interaction sessions, and automated parameter tuning to support personalized rehabilitation strategies. These extensions are expected to improve the system’s adaptability and extend its potential use in real clinical environments.

## Figures and Tables

**Table 1:** Hand and wrist movements enabled by the Omega 7.

Movement	Description
Finger Flexion and Extension	The device supports precise control over finger movements, improving mobility and strength in the fingers, which is essential for tasks like typing or holding objects.
Wrist Flexion and Extension	By simulating natural wrist movements, the Omega 7 helps restore wrist control and flexibility, critical for daily activities such as lifting or pouring.
Pronation and Supination	The device facilitates rotational forearm exercises, enabling patients to practice movements like turning a key or using a screwdriver, which are often affected by rigidity in PD and AD.
Radial and Ulnar Deviation	These lateral wrist movements are strengthened through targeted exercises, improving stability and control for tasks that require side-to-side hand motion.
Pinch Exercises (Tip, Key, and Palmar Pinch)	The Omega 7’s grip interface allows for precise training of fine motor skills and grip strength, essential for activities like buttoning a shirt or picking up small objects.
Grasp and Release	The device trains functional hand use by simulating real-life grasping and releasing motions, such as holding a cup or releasing a doorknob.
Active-Assistive and Resistive Movements	The Omega 7 can provide both assistance and resistance during exercises, supporting progressive rehabilitation tailored to the patient’s current abilities.

## Data Availability

The original contributions presented in this study are included in the article/Supplementary Materials. Further inquiries can be directed to the corresponding author.

## Supplementary Materials

The following supporting information can be downloaded at: https://www.mdpi.com/article/10.3390/bioengineering12121340/s1. Additionally, in Supplementary Data a video recording illustrating the testing of the Sudoku-based application is uploaded.

## Abbreviations

The following abbreviations are used in this manuscript:

AD	Alzheimer’s Disease
CSLA	Cognitively Stimulating Leisure Activities
DOF	Degrees of Freedom
FFT	Fast Fourier Transform
PD	Parkinson’s Disease
SD	Standard Deviation
WPD	Wavelet Packet Decomposition
