# Supplementary material for: Technical Validation of a Multimodal Cognitive—Haptic Sudoku Platform Under Simulated Tremor Conditions
Source: Bioengineering (Basel). 2025 Dec 9;12(12):1340. doi: 10.3390/bioengineering12121340 (PMC12729317; doi:10.3390/bioengineering12121340)
Supplement: Supplementary file 1 [file bioengineering-12-01340-s001.zip › IRB_Application_Sudoku_signed.pdf]

IRB Application

Study Title

Interactive Haptic Application for Fine Motor Evaluation in Healthy Participants – Exploratory, Preclinical Study

1. Principal Investigator (PI)

Name: Drd. Ing. Gabriela RUS
Department: Research Center for Industrial Robots Simulation and Testing
Email: Gabriela.Rus@mep.utcluj.ro

2. Purpose of Study

To evaluate an interactive puzzle-based application (inspired by Sudoku), controlled via a haptic device, aimed at fine motor skill engagement. The study serves as a preclinical exploratory stage for potential future rehabilitation use. This work will contribute to academic understanding of neurodegenerative diseases and could inform future clinical studies in rehabilitation. This study will serve both as academic research and, where applicable, fulfill educational degree requirements (e.g., thesis or capstone project).

3. Participant Description

Population: Healthy adult volunteers, recruited from the student body and broader university community.
Sample Size: 15 participants.
Inclusion Criteria:
- Ages 18–65
- No neurological or musculoskeletal conditions

Exclusion Criteria:
- Current upper limb injury
- History of epilepsy or visual impairments
- Undergoing treatment for mental health disorders that may be aggravated by visual illusion

4. Procedures

Location: B-dul Muncii 103-105, 400641 Cluj-Napoca
Each participant will attend one session lasting ~45 minutes.
Setup: Participants were invited to the lab and interacted with the application for approximately [45 minutes]. The task involved selecting and completing numeric cells using

the Omega 7 haptic device. No physical effort or risk was involved. No personally identifiable data was collected. The study did not involve any medical procedures or therapeutic interventions.
All tasks are low-intensity and seated.

All collected data are fully anonymized and cannot be linked to individual identities. Any recordings or screenshots were taken only with explicit consent. Each participant signed an informed consent form.

5. Risks

Risks are minimal:
- Mild muscle fatigue or discomfort
- Visual disorientation (rare)
- Confidentiality concerns (addressed below)

Mitigation:
- Clear explanation and rest breaks
- Right to withdraw at any time
- Emergency contact available during sessions

6. Benefits

No direct personal benefit
Educational value for students and scientific contribution to future rehabilitation research

7. Data Handling

Participants will be assigned random ID numbers
Data stored on encrypted, password-protected institutional drives
All identifiers removed before analysis
Data retained for 5 years then deleted securely

8. Informed Consent

Participants will receive:
- Information sheet (study purpose, procedures, risks, benefits, rights)
- Informed consent form (signature required)

They may ask questions at any time and can withdraw without consequences.

9. Compensation

No compensation

10. Conflict of Interest

No conflicts of interest exist.

11. Funding

No external funding

14. Ethical Compliance Statement

The study was conducted in accordance with the Declaration of Helsinki involving healthy subjects. The protocol was approved by the Ethics Committee of contract no. 760071/23.05.2023 on 02 June 2025 and can be reffered with the code: S01-02.06.2025.

15. Signatures and Approvals

The following signatures confirm institutional awareness and ethical compliance of the proposed research:

Principal Investigator (PI):
Drd. Ing. Gabriela RUS
Date: 02.06.2025

CESTER Supervisor:
Prof. Dr. Ing. Doina PISLA
Date: 02.06.2025

Program Co-Director:
Prof. Dr. Ing. Calin VAIDA
Date: 02.06.2025
